# Can Data Sharing Become the Path of Least Resistance?

**DOI:** 10.1371/journal.pmed.1001949

**Published:** 2016-01-26

**Authors:** The PLOS Medicine Editors

## Abstract

In this month’s editorial, the *PLOS Medicine* editors note recent progress towards data sharing as the community norm in medical research and the barriers that remain.

The year 2016 could be the year when medical research converges on data sharing as a universal standard, if recent events, reflected in several *PLOS Medicine* articles this month, are a good indication. Attaining that standard, however, may take a little longer.

Even in morally straightforward cases, data sharing can encounter roadblocks, as discussed in a recent Policy Forum by Vasee Moorthy and colleagues at the World Health Organization [1]. WHO convened a consultation in September, inviting scientists, medical journal editors, representatives of industry, funding organizations, and government “[i]n recognition of the need to streamline mechanisms of data dissemination—globally and in as close to real-time as possible” in the context of public health emergencies. Specifically, the consultation sought to prevent the kind of delays in data sharing that may have impeded resolution of the 2014–2015 Ebola crisis. Editors attending the consultation—representing *BMJ*, Nature journals, *New England Journal of Medicine*, and the PLOS journals—were called on to address a concern that data sharing in an emergency could lead to subsequent rejection of research by journals, on the grounds of prior publication.

The editors responded with a consensus statement agreeing that journal policies should not hinder data sharing to mitigate global public health emergencies: “In such scenarios, journals should not penalize, and, indeed, should encourage or mandate public sharing of relevant data" [2]. A subsequent Comment in *The Lancet* expressed support for data sharing in public health emergencies by authors from major research funding organizations [3]. The International Committee of Medical Journal Editors (ICMJE), meeting in November 2015, lent further support to the principles of the WHO consultation by amending ICMJE "Recommendations" [4] to endorse data sharing for public health emergencies of any geographic scope: “In the event of a public health emergency (as defined by public health officials), information with immediate implications for public health should be disseminated without concern that this will preclude subsequent consideration for publication in a journal.”

Public health emergencies present an ethical imperative to share data from studies of all kinds. Clinical trials, which involve an obligation to make the most of information obtained from volunteers who assume the risk of participation—particularly when this information which may affect the health of much wider groups—provide another example of ongoing progress in data sharing. After considering issues of particular relevance to clinical trials, including topics presented in the Institute of Medicine report of January 2015 [5], editors of the 14 ICMJE member journals, including *PLOS Medicine*, jointly published a proposed plan last week that, if adopted following public comment, will require data sharing for all clinical trials that will be published in the many journals that endorse ICMJE recommendations [6]. The plan would provide a major impetus for sharing data, reminiscent of the 2004 ICMJE requirement for registration of clinical trials [7]. Implementation would mean that researchers wishing to publish clinical trials in any of the large number of participating journals, including some of the most influential venues for clinical trial reports, would need to work with their institutional ethics committees to ensure that, going forward, informed consent language permits sharing of deidentified individual participant data (IPD).

Since March 2014, all PLOS journals have required data to be shared at the time of publication for all types of research, including clinical trials, and will continue to require data sharing at the time of publication (which is consistent with the ICMJE proposal to require sharing no later than 6 months after journal publication). The PLOS data policy aims to advance sharing without delay while respecting prior agreements between researchers and study participants. The policy therefore allows limited exceptions, as journals cannot reasonably impose data sharing when trial participants have not agreed that their individual (deidentified) data may be shared. Changes in the language of future informed consent agreements to include data sharing, as indicated in the ICMJE proposal, would greatly reduce this barrier.

Beyond the efforts of journal editors, medical research funders and industry sponsors have instituted advances in data sharing. For example, The Bill & Melinda Gates Foundation launched an open access policy in January 2015 including a requirement that data underlying their grantees’ published research be made accessible and open [8]. During a 2-year transition period, this policy has permitted a 12-month embargo, but as of January 2017 immediate sharing will take effect. The pharmaceutical company GlaxoSmithKline (GSK) has implemented a progressive data sharing policy for its clinical trials and has been joined by several other companies that now provide access to study data [9]. In a recent *PLOS Medicine* Perspective, Patrick Vallance and colleagues at GSK affirm that “for clinical trial data in particular the case for sharing patient level data is compelling whether it be to use the data from a number of studies to ask new questions and avoid waste, aggregate data to improve the evidence base for medicine, improve clinical trial design, or reduce unnecessary exposure of patients to risks in potentially futile clinical trials” [10].

Despite progress on several fronts, substantial challenges to data sharing remain. These include ensuring the quality of urgently disseminated data and efficiently sharing data across disparate platforms [1]; implications of data sharing for products undergoing regulatory review processes [10]; burdens and costs associated with preparing IPD and associated documentation for sharing, and potential for invalid analyses [11]; and maintaining confidentiality of study participants. Nor is it always a simple matter to define what data should be shared, as Deborah Zarin and Tony Tse illustrate in a new *PLOS Medicine* Essay based on their experience at the registry and results database ClinicalTrials.gov [11]. While IPD is typically seen as the most definitive information to be shared in clinical research, Zarin and Tse show how the term "IPD" in reality includes data at various stages of refinement, transformation, and aggregation, ranging from raw, uncoded data to extensively analyzed and summarized outcomes; which form is most useful to share depends on the question to be answered. Moreover, mandating sharing of only the IPD underlying a specific journal article is insufficient to prevent bias resulting from selectively unpublished trials; addressing this issue requires prior registration of all clinical trials. IPD sharing is thus most effective within the context of a “trial reporting system,” designed to increase the transparency of clinical trials systematically, which encompasses prospective trial registration and summary results reporting in public registries as well as IPD sharing [11].

Not the least among challenges will be ensuring due credit for analyses of shared data, so that groups with greater speed or resources for conducting analyses do not gain unduly in reputation at the expense of researchers who share data that they have dedicated their own resources to obtain. While this theme recurs throughout these recent articles, none proposes a specific answer, perhaps because any viable solution necessarily requires action by the clinical research community as a whole. The ICMJE proposal puts this issue directly to the community:

“…those who generate and then share clinical trial data sets deserve substantial credit for their efforts. Those using data collected by others should seek collaboration with those who collected the data. However, because collaboration will not always be possible, practical, or desired, an alternative means of providing appropriate credit needs to be developed and recognized in the academic community. We welcome ideas about how to provide such credit” [6].

Engagement with this issue will be crucial, because as long as authorship of individual published reports is perceived to confer greater reward than generating and sharing the data that underlie them, a disincentive to share data will persist.

Large and complex organizations and the structures that support them have started changing in order to reduce barriers to data sharing in medical research. The pieces are falling into place with such rapidity that opposing the sharing of data may soon find as little overt support within the research community as opposing the theory of gravity. However, just as a fundamental force can be attenuated by frictional drag, data sharing will not advance freely until participants across the research enterprise apply their insight and imagination to address the remaining reasons for resistance.

## Abbreviations

GSK: GlaxoSmithKline
ICMJE: The International Committee of Medical Journal Editors
IPD: individual participant data
